# Constructor theory of life

**DOI:** 10.1098/rsif.2014.1226

**Published:** 2015-03-06

**Authors:** Chiara Marletto

**Affiliations:** Department of Materials, University of Oxford, Oxford OX1 6UP, UK

**Keywords:** neo-Darwinian evolutionary theory, quantum theory, constructor theory, replication and self-reproduction

## Abstract

Neo-Darwinian evolutionary theory explains how the appearance of purposive design in the adaptations of living organisms can have come about without their intentionally being designed. The explanation relies crucially on the possibility of certain *physical processes*: mainly, *gene replication* and *natural selection*. In this paper, I show that for those processes to be possible without the *design of biological adaptations* being encoded in the laws of physics, those laws must have certain other properties. The theory of what these properties are is not part of evolution theory proper, yet without it the neo-Darwinian theory does not fully achieve its purpose of explaining the appearance of design. To this end, I apply constructor theory's new mode of explanation to express exactly within physics the appearance of design, no-design laws, and the logic of self-reproduction and natural selection. I conclude that self-reproduction, replication and natural selection are possible under no-design laws, the only non-trivial condition being that they allow *digital information* to be physically instantiated. This has an exact characterization in the constructor theory of information. I also show that under no-design laws an accurate replicator *requires* the existence of a ‘vehicle’ constituting, together with the replicator, a self-reproducer.

## 1. Introduction

Living entities display regularities unlike those observed in any other kind of matter. Although regular shapes of planets or crystals can be striking, these are explained by symmetries in the laws of physics; by contrast, even simple organisms, such as bacteria, display stupendously designed mechanisms, with *many, different sub-parts coordinating to an overall function*; they perform transformations with remarkable accuracy, retaining their ability to do so—just as if they had literally been designed. This *appearance of design* is the characteristic property of life relevant in this paper: the word *life* shall therefore be used to designate objects with that property.^1^

The appearance of design was long considered evidence of intentional design [1–3]: Why is it there? How did it come into existence? The theory of evolution [4] explains how the appearance of design can have been brought about by an undesigned physical process of variation and natural selection. It is a principle of this theory that everything with the appearance of design must have come into existence by natural selection—directly (e.g. organisms) or indirectly (objects that have literally been designed, e.g. cars or robots).

In the neo-Darwinian synthesis [5–7], the centrepiece of the explanation is a *physical object*—the *replicator* [5]: something that can be copied from generation to generation, by *replication*, and *selected* (between a set of variants) under the environment's action. Instances of replicators in the Earth's biosphere are ‘genes’, i.e. portions of certain DNA molecules.^2^ Natural selection relies on gene replication, with occasional errors; the appearance of design is explained as adaptations for gene replication; and the rest of the cell or organism (and sometimes other parts of the environment, e.g. nests, [6]) constitutes a *vehicle* for the replicators.

Thus, the neo-Darwinian evolutionary theory relies on the laws of physics to permit replication and the processes essential to the latter—including, as I shall explain, *self-reproduction*. Therefore, for the theory to explain fully the appearance of design, it is essential that those processes be *possible under laws of physics that do not contain the design of biological adaptations*—here called *no-design* laws.^3^

In this paper, I show that those processes are possible, provided that those laws have certain other properties. Although the theory of what these properties are does not belong to evolutionary theory proper, the neo-Darwinian theory cannot fully explain the appearance of design without it.

To explain why, let us examine the physical processes central to evolutionary theory. A replicator is an object *R* (in a set of variants) capable of undergoing *replication*, i.e. of being copied in this schematic pattern:


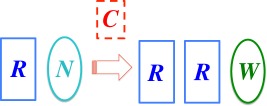


*C* is the copier, acting on raw materials *N* (possibly producing waste products *W*). For looser replicators, such as crystals, or short RNA strands and autocatalytic cycles involved in the origin of life, [9], the copier *C* is *null*, i.e. implicit in the laws of physics. For more accurate replicators, e.g. cellular DNA, the copier is in the cell, whose self-reproduction is essential to replication over many generations.

A *self-reproducer* is an object *S* capable of undergoing the physical transformation


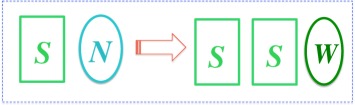


where the raw materials *N* need not contain the means to create another *S*, and the whole system could be isolated. Here it suffices to model the logic of self-reproduction only as it occurs in early life and pre-life—for instance, attention can be confined to unicellular organisms reproducing asexually in a non-biological environment. In that context, the difference between a replicator and a self-reproducer is that while a replicator *is allowed* to use a copier *C* outside itself, a self-reproducer *must not* rely on any mechanism other than itself and the laws of physics to produce the new instance. As I shall explain, under no-design laws an *inaccurate* self-reproducer (e.g. the ones involved in the origin of life) *may* consist of a replicator only, whereas an *accurate* self-reproducer (such as a bacterial cell) *must* consist of a replicator and a vehicle. In the latter case, self-reproduction occurs by *copying* the replicator and re-constructing the vehicle (including the replicator's copier *C*) afresh. This ‘replicator–vehicle’ logic (see §3.2) was discovered by von Neumann [10], and its relevance in biology analysed in [7,11].

In the biosphere self-reproduction is approximated to various accuracies. Pre-biotic crude replicators (e.g. short RNA strands) are poor approximations to self-reproducers. Being so inaccurate, they do not require any further explanation under no-design laws: they do not have the appearance of design, any more than simple inorganic catalysts do.^4^

By contrast, a bacterial cell can self-reproduce to high accuracy in a variety of environments, reconstructing the vehicle afresh, under the control of the genes, in all the details necessary for gene replication; and the latter is impressively accurate, albeit imperfect. This is *prima facie* problematic under no-design laws: how can those processes be so accurate, without their design being encoded in the laws of physics? Thus some physicists—notably, Wigner [12] and Bohm [13]—have even claimed that *accurate* self-reproduction of an organism with the appearance of design requires the laws of motion to be ‘tailored’ for the purpose—i.e. containing its design.

These claims, stemming from the tradition of incredulity that life can be scientifically explained, [14], highlight a problem. The theory of evolution must be supplemented by a theory that those physical processes upon which it relies are provably compatible with no-design laws.

No such theory has been proposed; nor have those claims been properly refuted. Indeed, the central problem—i.e. *whether and under what circumstances accurate self-reproduction and replication are compatible with no-design laws*—is awkward to formulate in the *prevailing conception of fundamental physics*, which expresses everything in terms of predictions given some initial conditions and laws of motion. This mode of explanation can only approximately express emergent notions such as the appearance of design, no-design laws, etc.

In this framework, von Neumann discovered the essential (replicator–vehicle) logic of self-reproduction, [10]. However, using the prevailing conception forced his analysis to be in terms of predictions: thus, he attempted without success to provide the design of an actual self-reproducer in terms of atoms and microscopic interactions. He finally produced a viable toy model, [15], within cellular automata, but at the cost of severing the connections with actual physics. Thus, that model cannot address the current problem—whether self-reproduction is compatible *with the actual laws of physics* un-augmented by any design of adaptations.

The prevailing conception also forces a misleading formulation of the problem as: what initial conditions and laws of motion *must* (or must probably) produce accurate replicators and self-reproducers? But what is disputed is whether such entities are *possible* under no-design laws.^5^

The prevailing conception cannot express the very explanation provided by evolutionary theory—that living organisms *can* have come about without intentionally being designed. At best, it would aim at proving that they *must* (or must probably) occur, given certain initial conditions and dynamical laws.

To overcome these problems, I resort to a newly proposed physical theory, constructor theory [18–20]. It provides a new mode of explanation, expressing all laws as statements about which transformations are *possible*, which are *impossible* and why.

This brings *counterfactual statements* into fundamental physics, which is key to the solution. The theory of evolution is already constructor-theoretic: it is *possible* that the appearance of design has been brought about without intentionally being designed. So is our problem: are the physical processes essential to the theory of evolution—i.e. self-reproduction and replication—*possible* under *no-design* laws?

I shall show that they are (in §2–3) provided that those laws allow the existence of media that can instantiate (digital) *information* (plus enough time and energy). *Information* is characterized *exactly* in the constructor theory of information [19].

I also show that under no-design laws an accurate self-reproducer *requires* a *high-fidelity* replicator, and *vice versa*. Thus, von Neumann's replicator–vehicle logic is shown to be *necessary* for accurate self-reproduction under such laws. This provides a physical foundation for the dichotomy between replication and ‘metabolism’ (as introduced by Dyson [11]). In addition, that vehicles are necessary to high-quality replicators under our laws of physics—despite replicators being the conceptual pillar of evolutionary theory—informs the debate about the necessity of organisms. The latter was recently doubted by Dawkins [21]: ‘Living materials did not have to become packaged into discrete, individual organisms […] behaving as unitary, purposeful agents. The only thing that is really fundamental to Darwinian life is self-replicating, coded information—genes’.

Constructor theory also delivers an exact physical expression of the appearance of design, no-design laws and of the *logic* of self-reproduction and natural selection.^6^

Finally, Wigner's argument implies that accurate self-reproduction is incompatible particularly with *quantum theory*, thus challenging the latter's universality—a claim that others, with different motivations, have also made [22–24]. I shall demonstrate (in §4) a quantum-mechanical (kinematical) model of the *logic* of self-reproduction, updating von Neumann's, thus rebutting those claims. This, incidentally, clarifies how self-reproduction differs from cloning a quantum state (which has occasionally caused some confusion [22]). It also vindicates that self-reproduction—and even (possibly artificial) self-reproducers employing quantum coherence—are compatible with quantum theory.

## 2. The problem

I shall introduce constructor theory to re-formulate the problem in constructor-theoretic terms.

Constructor theory is a new fundamental theory of physics. First, it provides a paradigm where the other laws of physics are expressed *solely* as statements about which transformations are *possible*, which are *impossible* and why. Guesses at those laws—e.g. general relativity and quantum mechanics—it calls *subsidiary theories*. In addition, it also proposes new laws, principles, constraining the subsidiary theories. Here it suffices to know that those principles are obeyed by all known laws of physics, nor do they themselves contain the design of biological adaptations [18,19].

The properties of a physical system **M** are *attributes*, defined as sets of states of **M**. The system **M** (say, a collection of atoms) has the attribute *X* (say, being a car, or a self-reproducer) if it is in any of the states in *X*.

Constructor theory's main elements are *tasks*. A *task* *T* is the abstract specification of a transformation
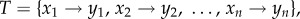
as a *set of input/output pairs* of attributes {*x_i_*}, {*y_i_*} of the *substrates* (the physical systems being transformed).

Tasks form an algebra under parallel and serial composition and are composable into networks to form other tasks [19].

A physical system with some attribute 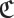 is *a constructor*, *capable of performing the task T* if

— whenever presented with the substrates with any of the legitimate input attributes of *T*, 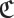 delivers it with the corresponding output attribute, as follows
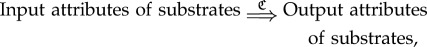
where 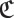 and the substrates *jointly constitute an isolated system*;
— 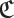 retains the ability to do so again.

A task is *impossible* if it is forbidden by the laws of physics (e.g. building a perpetual motion machine); otherwise, it is *possible*.

Under our laws of physics, only approximate constructors exist, e.g. catalysts or robots. They have non-zero error rates and deteriorate with use. Hence, that a task is possible means that the laws of physics impose no limit, short of perfection, on how accurately it could be performed, nor on how well objects capable of approximately performing it could retain their ability to do so. The term ‘constructor’ is a placeholder for the (infinite) sequence of approximations to the ideal behaviour of a constructor.

Both replication and self-reproduction are expressed exactly in constructor theory. The *replication* of a set *Σ* of attributes is the task2.1
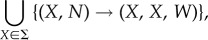
on the composite system **M**_1_ ⊕ **M**_2_ of the *source* substrate (containing the attribute to be copied) and the *target* substrates (onto which the attribute is copied). 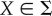 is an attribute of **M**_1_, being replicated; *N* some receptive attribute of **M**_2_ and (*X*, *W*) the output attribute, including waste products *W*. For example, *Σ* could be a set of musical notes; or the set of alleles (variants of a gene) or the set of nucleotides.^7^

*Self-reproduction* is a *construction* where the self-reproducer *S* is a constructor for constructing another instance of itself given raw materials *N* containing neither *S* nor constructors for *S*2.2
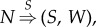
allowing for waste products. *S* is the specification of all properties necessary for (2.2) to occur, given the laws of physics.

*No-design laws* can be expressed exactly in constructor theory, too.

First, I define *generic resources* as substrates that exist in *effectively unlimited* numbers. In the context of early life on the Earth, these include only elementary entities such as photons, water, simple catalysts and small organic molecules.

It has sometimes been proposed that the very existence of laws of nature constitutes a form of ‘design’ in them, [25]. By contrast, here no-design laws are those that do not contain the design of what the theory of evolution aims at explaining—i.e. biological adaptations. Thus, I require them to satisfy these conditions

— Generic resources can only perform *a few* tasks, only to a finite accuracy, called *elementary tasks*. These are physically simple and contain no design (of biological adaptations). Examples are spontaneous, approximately self-correcting chemical reactions, such as molecules ‘snapping’ into a catalyst regardless of any original small mismatch.
— No good approximation to a constructor for tasks that are non-elementary can ever be produced by generic resources acting on generic resources *only*.

So, under no-design laws, generic resources and the available physical interactions are allowed to contain only approximate constructors—unequivocally not bearing the design of the adaptations explained by evolutionary theory.^8^ Examples of laws violating these conditions are: laws where generic resources include accurate constructors, such as bacteria; laws whose interactions are designed to copy the configuration of atoms of a bacterium onto generic resources; laws permitting spontaneous generation of a bacterium *directly* from generic resources only; laws permitting only mutations that are systematically directed to *improvements* in a certain environment.

Characterizing no-design laws exactly is a departure from the prevailing conception—which can at most characterize them as being typical, according to some measure, in the space of all laws. This approach is unsuitable for present purposes, as the choice of the measure is arbitrary. Moreover, some laws that may be untypical under some natural measure (such as the actual laws of physics, because of, say, local interactions) may qualify as no-design in this context—they need not contain the design of adaptations. Specifically, in the context of anthropic fine-tuning, one calls ‘bio-friendly’ laws with features (e.g. local interactions, the value the fine-structure constant) that if slightly changed, would make life impossible; But these laws may still be no-design, because those features are *not specific to life*: their variation would make impossible many other phenomena, non-specifically related to biological adaptations.

The problem can now be restated in constructor theory, as: *are accurate self-reproducers and replicators possible under no-design laws?*

I shall prove that an accurate self-reproducer is possible under no-design laws, provided they allow information to be physically instantiated; from this it will follow that an accurate replicator is possible too, provided that it be contained in a self-reproducer, (§§3.1–3.3).

I will assume that the raw materials of self-reproduction (*N* in (2.1), (2.2)) comprises generic substrates *only*. This over-stringent assumption—ruling out the realistic situation that they contain other organisms—suffices for present purposes: if accurate self-reproduction and replication are allowed under these requirements, so are they when the generic resources contain also living organisms.

I shall now recall the basics of the constructor theory of information (§2.1) necessary to express what it means for the laws of physics to permit information to be physically instantiated.

### 2.1. Information

Replication, regarded as copying, is intimately connected with *information*. This has inspired some information-based approaches to fundamental problems in biology [26].

The connection between information and physics, specifically thermodynamics, has been known for some time, e.g. [27]. However, until recently, information had no place in fundamental physics, being considered as an inherently approximate notion. For example, expressions such as ‘information being physically instantiated’ did not have a precise physical meaning. But the *constructor theory of information* has now incorporated information within fundamental physics [19], providing an exact physical characterization to those expressions, as follows.

A set of attributes *Σ* is an *information variable* [19] if the task of performing any *permutation* over *Σ* (allowing for waste), and the *replication task* over *Σ*, as in (2.1), are all possible. The attributes of an information variable are called *information attributes*. An *information medium* is a substrate some of whose attributes constitute an information variable.

Information media must obey the *interoperability principle* [19]: the composite system of two information media with information variables *Σ*_1_ and *Σ*_2_, is an information medium with information variable *Σ*_1_ × *Σ*_2_. This is a physical principle, requiring there to be interactions such that information is ‘copiable’ from one information medium to any other.

Thus, whether or not information media exist, i.e. whether or not information can be instantiated in physical systems, depends on the laws of physics. The connection between replication and information is expressed as a physical law: dynamical laws permitting information media must allow information variables—i.e. *replicable* sets of attributes as in (2.1).

A physical system **M** *instantiates information* if it is an information medium in one of its information attributes (belonging to some information variable *Σ*) and if the task of giving it any other attribute in *Σ* (allowing for waste) is possible. This intrinsic, *counterfactual*, property of **M** is an exact physical requirement, that certain interactions be available in nature.

A constructor *C* for the replication task on some information variable *Σ*
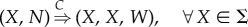
is called a *copier* of *Σ*.

Of its substrates, one—the *target*—is changed from having the attribute *N* to having the attribute (*X*, *W*); the other—the *source*, initially having one of the attributes *X* in *Σ*, to be replicated—remains *unchanged* (but may change temporarily during the copying).

**Figure 1.**
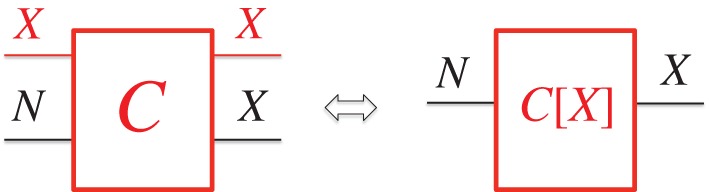
Two representations of a copier *C* (waste *W* omitted). On the left, *C* is a constructor, with substrates represented by lines: the source, remaining unchanged; and the target, that is changed. On the right, *C* and the source substrate with attribute *X* constitute the constructor *C*[*X*] performing the task *T_X_* = {*N*→*X*}.

Therefore, *C* and the source with attribute *X* constitute a constructor *C*[*X*] performing the task *T_X_* = {*N*→(*X*, *W*)} on the target. The information attribute *X* in the source acts as a constructor, *instructing C* to perform the task *T_X_* on *N* (figure 1).

In general, a *programmable constructor* is a constructor *V* one of whose input substrates is an information medium **M** holding an information attribute *P*, with the property that **M** (with that attribute) is itself a constructor. *V*[*P*] is a constructor for the task *T_P_*, *P* is the program for the task *T_P_* and *T_P_* is in the repertoire of *V*. For example, *V* could be the ribosome, *P* the sequence that, when inserted in *V*, would cause *V* to perform the task *T_P_* of constructing a particular polypeptide chain. The information instantiated in *P* is an *abstract constructor*—an instance of ‘information with causal power’ [28].

## 3. The theory of evolution by natural selection is compatible with no-design laws

I shall now show that under no-design laws accurate self-reproducers and replicators are not forbidden, provided only that the laws permit the existence of information media (and enough resources). This will vindicate that the theory of evolution by natural selection is compatible with those laws (and thus, in particular, with the current theories of physics).

My argument includes three steps. First, I establish that an accurate constructor for a generic task is compatible with no-design laws (§3.1), provided that it contains a replicator, instantiating a recipe for that task. As a special case, I show that accurate self-reproducers are compatible with no-design laws (§3.2), provided that they implement the ‘replicator–vehicle’ logic; it follows that so are accurate replicators, and that they require a self-reproducer. Finally, I show that the logic of natural selection is compatible with no-design laws (§3.3).

### 3.1. An accurate constructor must contain a replicator

A task *T* being possible means that for any given accuracy (short of perfection) the laws of physics permit an approximate constructor capable of performing the task to that accuracy.

Consider a possible, non-elementary task *T* and an object *F* that can perform *T* to a high accuracy *ε*.^9^ For instance, *T* could be the task of constructing a car from generic substrates and *F* a generalized car factory, including all the processes converting raw materials, such as iron, etc., into a car.

The approximate constructor *F executes* a procedure—a *recipe*—to perform the task *T* to accuracy *ε*. I will show that *F* must include a replicator and a programmable constructor; and that the recipe must have a hierarchical structure and be instantiated in the replicator.

No-design laws contain no good constructor for *T*, such as *F*—neither in the elementary interactions, nor in the generic resources. Hence, the *recipe* used by *F* to perform *T* must be decomposable into steps (not necessarily sequential) that are allowed by no-design laws, i.e. *sub-recipes*—procedures to perform sub-tasks that are executed by sub-constructors contained in *F*. To avoid infinite regress, two conditions must be fulfilled.

One is that the sub-tasks be *non-specific* to *T*. For instance, when *T* is the task of constructing a car, the sub-tasks are those of constructing sub-parts of the car—e.g. door handles, windows, etc. Hence, the constructor *F* must include two parts: one—called *V*—performs *T blindly*, i.e. subtask by subtask, and it is non-specific to *T*, because so are the sub-tasks. The rest of *F*—called *P*—is specific to *T* and instantiates the recipe for *T*—the sequence of sub-tasks controlling *V*. Hence, *F* is a programmable constructor, *V*, programmed with a program *P* having *the same logic as the recipe*: it has a modular structure *P* = (*p*_1_, *p*_2_, … , *p_N_*) where each instruction *p_i_* takes values in an information variable and tells *V* which sub-task to perform, when, on the substrates^10^. *V* is non-specific to *T* because it must also be capable of executing other programs—different combinations of the elementary units *p_i_*. For example, a car factory contains robots executing sub-recipes to construct the car's doors. These robots contain sub-robots to construct handles, windows, etc., usable to construct other objects than cars.

The other condition is obtained by applying the same reasoning recursively to the sub-tasks. If they, too, are non-elementary, they require a recipe that is decomposable into non-specific sub-recipes. The base for the recursion—for *T* to be performable to that particular accuracy—is provided by the *elementary sub-recipes* of the recipe for *T* being elementary tasks—which can be performed by (approximations to) constructors that are available in nature, as generic resources.

These elementary sub-tasks need not be specified in the recipe: they are implicit in the laws of physics. For instance, the elementary steps in the car recipe are tasks like, say, ‘oxidize the aluminium coating’, occurring simply by leaving the substrate exposed to air.

Under no-design laws, any (approximation to a) constructor *wears out* after a finite time. Therefore *F*, to perform the task *T* to the accuracy *ε*, must undergo a process of *maintenance*, defined as one whereby a new instance of *F*—i.e. of *P* and *V*—is constructed, from generic materials, before the former one stops working. In the car factory, this is achieved by replacing old sub-parts of the robots, assembly lines, etc., and by preserving the programs they run.

To avoid an infinite regress, the maintenance must not in turn require the recipe *P* for *T*. Also, the recipe's design cannot be in the laws of physics. Thus, the only other possibility is that the new instance of the recipe is brought about by *blind replication* of the recipe contained in the former instance—i.e. by replicating its subunits *p_i_* (non-specific to *T*). We conclude that, under no-design laws, the recipe must necessarily be instantiated in a *modular replicator*: a physical object that can be copied blindly, an elementary subunit at a time. By contrast, *V*—the non-specific component of *F*—is *constructed* anew from generic resources.

Moreover, under no-design laws *errors* can occur: thus, to achieve high and improvable accuracy, the recipe must include *error-correction*. In the car factory, this includes, say, controlling the functionalities of the subcomponents (e.g. fine checks on the position of doors, wheels, etc.). Hence, *the recipe P must contain information about the task T, informing the criterion for error detection and correction*.

The information in the recipe is an abstract constructor that I shall call *knowledge* (without a knowing subject [29]). Knowledge has an exact characterization in constructor theory: it is information that can act as a constructor and cause itself to remain instantiated in physical substrates. Crucially, error-correcting the replication is necessary. Hence, the subunits *p_i_* must assume values in a *discrete* (digital) information variable: one whose attributes are separated by non-allowed attributes. For, if all values in a continuum were allowed, error-correction would be logically impossible.

#### 3.1.1. Appearance of design

Something with the appearance of design is often described as ‘improbable’ [30,31]. This is misleading because probability measures are multiplicative; so that two independent objects with the appearance of design would have much more of that appearance than they do separately. But that is not the case when the two objects have unrelated functionalities (such as, say, internal organs of different organisms). By contrast, two organs *in the context of the same* organism, coordinating to the effect of gene propagation, do have a greater appearance of design than either separately. Constructor theory expresses this naturally for programmable constructors.

Consider a recipe *R* for a possible task *T*. A sub-recipe *R*′ for the task *T*′ is *fine-tuned* to perform *T* if almost any slight change in *T*′ would cause *T* to be performed to a much lower accuracy. (For instance, changing the mechanism of insulin production in the pancreas even slightly would impair the overall task the organism performs.) A programmable constructor *V* whose repertoire includes *T has the appearance of design* if it can execute a recipe for *T* with a hierarchical structure including *several, different* sub-recipes, *fine-tuned* to perform *T*. Each fine-tuned sub-recipe is performed by a sub-constructor contained in *V*: the number of fine-tuned sub-recipes performable by *V* is a measure of *V*'s appearance of design. This constructor-theoretic definition is non-multiplicative, as desired.

### 3.2. The logic of self-reproduction

As I shall now argue, the results of §3.1 imply that no-design laws permit an *accurate* self-reproducer, such as a bacterial cell, provided that it implements what I call, adapting Dawkins' terminology [7], the *replicator–vehicle logic*.

A self-reproducer *S* (of the kind (2.2)) is a constructor for its own construction, from generic resources only. The argument in §3.1 implies that for *S* to be a good approximation to a constructor for another *S*, it must consist of: a modular *replicator*, *R* = (*r*_1_,*r*_2_,…, *r_n_*), instantiating the recipe for *S* (the elementary units *r_i_* have attributes in an information variable *Σ*, corresponding to instructions); a programmable constructor, the *vehicle V*, executing the recipe *blindly*, i.e. implementing non-specific sub-tasks.

The recipe instantiated by the replicator *R* must contain all the knowledge about how to construct *S*, specifying a procedure for its construction. However, the recipe is in one sense incomplete: as remarked in §3.1, it is not required to include instructions for the elementary tasks, which occur spontaneously in nature. These are indeed relied upon during actual cell development—they constitute epigenetics and environmental context. As remarked by George C. Williams, ‘Organisms, wherever possible, delegate jobs to useful spontaneous processes, much as a builder may temporarily let gravity hold things in place and let the wind disperse paint fumes’, [32].

Under no-design laws, maintenance and error-correction are necessary for a high and improvable accuracy to be achieved; in self-reproduction, crucially, it must be *S only* that produces the new instance of *S*. By the no-design condition, the maintenance cannot be performed by the laws of physics either: so it must be executed by *S*. As discussed in §3.1, maintenance must be achieved via *copying* the recipe and *constructing* the vehicle V. These actions are enacted, respectively, by two sub-constructors in the vehicle, *C* and *B*, implementing von Neumann's replicator–vehicle logic, [15].

In the *construction phase B* executes *R* to construct a new vehicle *V*
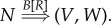


In bacteria *B* includes the mechanisms for constructing the daughter cell, such as the ribosome which uses DNA instructions (translated into RNA) to construct proteins. Blind error-correction can be performed by checking the sub-tasks; however, construction errors are not propagated, because the new vehicle is constructed following the recipe, *not* copying the former vehicle.

In the *copy phase*, *C*, the copier of the information variable *Σ*, performs the replication of *R*3.1
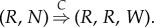


This entails replicating the *configuration* of *R blindly*, one elementary unit at a time. Hence, *C* is a universal copier for the set of replicators consisting of elementary units drawn from *Σ* (a property called *heredity* [33]). Error-correction can happen blindly too, for instance, via mismatch-repair. In bacteria this phase is DNA replication and *C* includes all the relevant enzymes in the cell.^11^ For the two phases to perform maintenance, the recipe for the vehicle *V*, instantiated in the replicator *R*, must be copied in the copy phase. This requires the elementary instructions of the recipe to be (sets of) the elementary units *r_i_* of the replicator. In bacteria they are the codons—triplets of the elementary units of the replicator (the nucleotides), coding for the building blocks of proteins (amino acids).

The replication of each sub-unit *r_i_* constitutes a *measurement* of which attribute *r_i_* holds, followed by constructing a new instance of it. Since the replicator *R* must contain all the knowledge about *S*, the attributes in *Σ*, of which *R* is made, must be *generic resources*, so as to require no recipe (other than *R*) to be constructed from generic resources. I call a modular replicator such as *R* whose subunits are made of generic resources a *template replicator*. A DNA strand is one: the information variable *Σ* is the set of nucleotides—they are simple enough to have been naturally occurring in pre-biological environments.

Thus the two maintenance phases achieve self-reproduction, as they amount to producing a new *R*, by copying the former *R*, and a new *V*, by construction controlled by *R*. Indeed, self-reproduction is stable precisely because copying and construction automatically execute the maintenance of *S*, by replicating the recipe and re-constructing the vehicle before the former instance of *S* wears out; and they permit error-correction. For arbitrarily high accuracy, both phases implement elementary, non-specific sub-recipes bearing no design. Therefore, arbitrarily accurate self-reproduction is permitted by no-design laws, provided that the latter allow replicators—i.e. information media.

**Figure 2.**
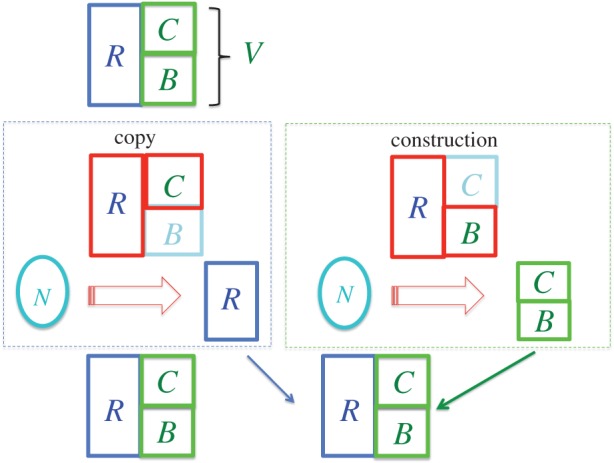
The logic of self-reproduction. An *accurate* self-reproducer (top) comprises the replicator *R* (blue outline) and the vehicle *V* (green outline)—containing the copier *C* and the constructor *B*. In the copy phase, *C* copies the replicator *R* − *C*[*R*] (red outline) acts as a constructor. In the construction phase, *B* executes the recipe in *R* to build a vehicle from generic resources *N* − *B*[*R*] (red outline) acts as a constructor. Finally (bottom), the copy of *R* and the newly constructed vehicle form the offspring.

Rewriting the copy phase, (3.1), as
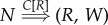
to highlight that *C* executes *R*, we see that a template replicator has a special property. It instantiates *a recipe for its own construction from generic resources only* (*C* does not need to contain any additional recipe to construct the subunits of *R*: it blindly copies the pattern, subunit by subunit; and the units are generic resources). This is unique to template replicators. No other object could be a recipe for the construction of itself to a high accuracy. For, by the argument in §3.1, an instance (or a blueprint) of an object is not, in general, a recipe for its construction from generic substrates. A three-dimensional raster-scanner provided with an instance of, say, a bacterium could not reproduce it accurately from generic substrates *only*: without a recipe containing the knowledge about the bacterium's structure, there would be no criterion for error-correction, resulting in a bound on the achievable accuracy. Likewise, an entire organism could not self-reproduce to a high accuracy via self-copying: without the recipe informing error-correction, an ‘error catastrophe’ [34] would occur. This also provides unifying descriptions of the two phases: the replicator *R* is a recipe for another instance of itself, when instructing *C*; a recipe for the construction of another vehicle, when instructing *B*. Overall, it instantiates *the full recipe for S* (figure 2).

*R* is an *active*, *germ line* replicator [7], because it instantiates all the knowledge *necessary to achieve its own replication*. It is a consequence of the above argument that high-fidelity replication is possible under no-design laws too, provided that there is a vehicle that performs blind copying and error-correction. Moreover, for the replicator to preserve its ability to be an accurate replicator across generations, its vehicle must be reproduced too—together, they must constitute a self-reproducer. Hence self-reproduction is essential to high-fidelity replication under no-design laws.

### 3.3. Natural selection is permitted under no-design laws

These conclusions imply that an accurate self-reproducer—together with an accurate replicator—is permitted under no-design laws permitting information media. So, under such laws, it can be constructed from generic resources only, given *enough knowledge*: it could continue to exist, say, had a chemical laboratory created it.

However, one must also address the question: *can* accurate self-reproducers arise from generic resources only, under such laws?

Note that the prevailing conception would aim to prove that the emergence of accurate self-reproducers *follows* (with some probability) given certain initial conditions and laws of motion. This approach, informing the search for models for the origin of life [28] is suitable to address problems such as predicting the existence of life elsewhere in the universe—e.g. by providing bounds to how probable the emergence of those self-reproducers is on an Earth-like planet. Here I address a different problem: whether accurate self-reproducers are *possible* under no-design laws. This is a theoretical (constructor-theoretic) question and can be addressed without resorting to predictions. Indeed, the theory of evolution provides a positive answer to that question, provided that two further points are established. I shall now argue for them.

*The first point* is that *the logic of evolution by natural selection is compatible with no-design* laws because—in short—selection and variation are *non-specific* to its end products. This is explicated by modelling the *logic* of natural selection as an approximate *construction*, whose substrates are populations of replicators and whose (highly approximate) constructor is the environment. Evolution relies upon populations being changed by variation and selection over the timescale spanning many generations: replicators—constructors for self-reproduction, on the shorter timescale—become now substrates. Crucially, the mutations *in the replicators*, caused by the environment, are non-specific, (as in §3.1), to the ‘end product’ of evolution (as Dawkins put it, not ‘systematically directed to improvement’ [30]). This constructor-theoretic characterization of mutations replaces the less precise locution ‘random mutations’ (as opposed to non-random selection, [5]). These mutations are *all* transmitted to the successfully created individuals of the next generation, by heredity—irrespective of their being harmful, neutral or beneficial in that particular environment.

Selection emerges from the interaction between the replicators and the environment with *finite* resources. It *may* lead to equilibrium, given enough time and energy. If so, the surviving replicators are near a local maximum of effectiveness at being replicated in that environment.

Thus, the environment is *passive* and *blind* in this process. As it retains its ability to cause non-specific variation and passive selection again, it qualifies as a naturally occurring approximation to a constructor. Crucially, it is a crude approximation to a constructor: crude enough that it could have arisen by chance and requires no explanation. Its actions—variations and selection—require no design in laws of physics, as they proceed by non-specific, elementary steps. Indeed, such processes are highly faulty constructions that produce, aside from knowledge, many waste products. So, the logic of evolution by natural selection is compatible with no-design laws of physics.

*The second point* is that natural selection, *to get started*, does not require accurate self-reproducers (with high-fidelity replicators and vehicles). Indeed, the minimal requirement for natural selection is that each kind of replicator produce *at least one viable offspring*, on average, per lifetime—so that the different kinds of replicators last long enough to be ‘selected’ by the environment. In challenging environments, a vehicle with many functionalities is needed to meet this requirement. But in unchallenging ones (i.e. sufficiently unchanging and resource-rich, such as pre-biotic ones), the requirement is easily met by inaccurate self-reproducers, without a vehicle. These not only have no appearance of design, but also are so inaccurate that they *can* have arisen spontaneously from generic resources under no-design laws—as proposed, for instance, by the current theories of the origin of life [9,35]. For example, template replicators, such as short RNA strands [33], or similar ‘naked’ replicators (replicating *with poor copying fidelity*, without a vehicle) would suffice to get natural selection started.^12^ Since they bear no design, they require no further explanation—any more than simple inorganic catalysts do.

I conclude that the theory of evolution is compatible with no-design laws of physics, that allow, in addition to enough time and energy, information media. These requirements do not contain the design of biological adaptations. Hence, under such laws, the theory of evolution fully explains the appearance of design in living organisms, without their being intentionally designed.

## 4. Self-reproduction under quantum theory

I shall now show that accurate self-reproduction is compatible with quantum theory: after a critique of works claiming the opposite (§4.1), I demonstrate a quantum-mechanical (kinematical) model of the replicator–vehicle logic (§4.2).

### 4.1. Irrelevance of the incompatibility arguments

The first claim that self-reproduction is incompatible with quantum physics was made by Wigner [12]. Its agenda is to show that ‘the present laws of quantum mechanics will have to undergo modifications before they can be applied to the problems of life’ and they need to be complemented by ‘biotonic’ laws, containing the design of self-reproducers [12]. The proposed method to do that is showing that the unitary laws of quantum physics which cause arbitrarily accurate self-reproduction of an organism *S* constitute a vanishingly small fraction of all possible unitaries, *when S is a sufficiently specialized entity* (has the appearance of design).

In Wigner's model the substrates of self-reproduction consist of three parts: the parent self-reproducer; the substrates to be transformed into the new instance and the substrates to be transformed into waste. Correspondingly, the total Hilbert is modelled as 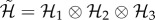, where the labels 1, 2, 3 refer, respectively, to those three parts, and 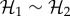 (I shall denote both by 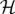). The ‘highly specialized’ self-reproducer is a subspace 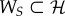 whose dimension *h*(*S*) is much smaller than the dimension *d* of 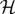. Wigner's argument shows that the set of unitaries causing the replication of *W_S_* in a given tensor-product structure
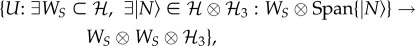
has measure which tends to zero as *h*(*S*)/*d* → 0 (with respect to the natural measure on the space of unitaries) [36].

Wigner concludes that unless the unitary *U* is ‘tailored so as to permit self-reproduction, it is infinitely unlikely’ that, under quantum theory, accurate self-reproduction of specialized entities can occur; whence the need for designed laws.

Evidently, this argument would not rule out self-reproduction only. It would apply to all the unitaries *U*: 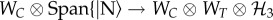 for some subspaces *W_C_*, *W_T_* whose dimension is smaller than *d*. Hence it would rule out, under Wigner's interpretation, *every* specialized construction.

But the interpretation is erroneous. As explained, the ‘non-typical’ interaction is compatible with no-design laws (and in particular with quantum mechanics, see §4.2), because it can be decomposed into elementary steps—non-specific to *S*—controlled by the recipe. No-design laws plus a knowledge-laden recipe can play the role that Wigner erroneously assumed can only be played by knowledge-laden laws and a generic initial state. Also, the ‘difficult feat’ [12] of bringing about the knowledge in the recipe does not require intentional design, as explained by evolutionary theory, which I showed is compatible with no-design laws.

The misconception underlying Wigner's interpretation is to identify the mathematical property of being a ‘non-typical’ unitary with the *physical* property of containing the design of an object. Evidently, the former does not imply the latter; so, the argument is irrelevant to the claim. Similarly, the (multiplicative) property of belonging to a small subspace misrepresents the appearance of design (which is non-multiplicative, see §3.1.1).

Moreover, as pointed out in [36], Wigner's *argument* is about an over-constrained set of unitaries, i.e. the ones causing reproduction of *W_S_* in a tensor-product structure that is fixed *a priori*. But Wigner's purpose is served by the set of unitaries with the property *that there exists* a tensor-product structure in which they would cause self-reproduction. Nevertheless, Baez's theorem, [36], that almost all unitaries would achieve replication of a *single state* in the presence of a *specific* initial state, in *some* tensor-product structure, is not actually a rebuttal of Wigner's *claim*. One could reach the same conclusion as Wigner's by arguing that this initial condition is in fact of zero-measure in the set of all possible initial conditions. Also, the replication of a single quantum state (which Wigner also discusses) is too strict a requirement to model self-reproduction of living entities, as it does not permit evolution.

Confusing self-reproduction and replication (cloning) of single quantum states has informed another claim, that self-reproduction of a universal constructor with finite resources is forbidden by quantum theory [22–24]. The model supporting this claim comprises a collection of substrates, with Hilbert space 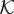, *n* of which are the raw materials, 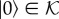; the rest contains the processor, the control unit and the program space of the alleged universal constructor. 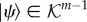 is any state of the processor and 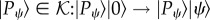 is the program for the state 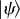. Self-reproduction of the universal constructor would correspond to a unitary satisfying, for some states 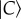, 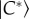 of the control unit
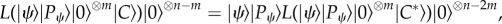
for every *ψ*. This is impossible, the argument goes, unless programs for different states are orthogonal; in which case (allegedly) infinitely many resources would be needed, as the program space would have to be infinite-dimensional. This claim, too, is irrelevant to whether living self-reproducers are compatible with quantum mechanics. *L* copies *each state* of the vehicle and the program for that state, while actual self-reproduction requires the re-production of a *subspace*—the property of being a self-reproducer. Indeed, *L* is ruled out by the no-cloning theorem, if the programs are not orthogonal. Besides, actual self-reproducers need not be universal constructors: their repertoire need only include (and, in the Earth's biosphere, does include) very few products, compared with the set of all possible products.

### 4.2. The replicator–vehicle logic under quantum theory

I shall now demonstrate a quantum-mechanical model of self-reproduction, implementing the replicator–vehicle logic. I model the world as a collection of replicas of the substrates that can have the attribute of being a self-reproducer. Each substrate has Hilbert space 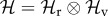, where 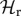 is the space of the replicator and 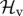 that of the vehicle. One replica contains the parent, one its offspring, and the remaining *w* are transformed into waste products. The law of motion is a unitary *U* which I shall prove to be compatible with self-reproduction. The attribute *A* is the +1-eigenspace of the projector 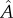 for holding that attribute: 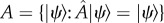.

Let 
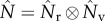
 (defined on 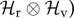 be the projector for being generic substrates and 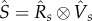 be that for being a self-reproducer *S*, where 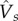 is the projector (defined on 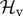) for being a vehicle and 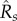 is the projector (defined on 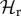) for being a recipe for it.

For evolution to be possible a set *Σ* of different self-reproducers must be allowed in the environment *N*. So, the unitary law of motion *U* must satisfy, 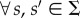
4.1
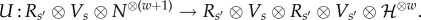


This is self-reproduction as in equation (2.2) (for *s* = *s*′); and each self-reproducer has heredity: a vehicle copies any recipe (coding for vehicles or not); and executes recipes to construct any other vehicle. Different vehicles are represented by mutually orthogonal projectors. By unitarity of (4.1), recipes for different self-reproducers are orthogonal too: 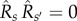. I shall confine attention to a basis of orthogonal programs spanning each subspace (their superpositions code, by linearity, for the same vehicle).

That the environment contains no knowledge about the self-reproducer is guaranteed by imposing the (sufficient) conditions 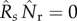, 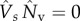, for all *s*.

Let 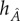 be the rank of the projector 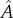, *d*_r_ the dimension of 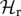, *d*_v_ that of 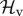. Each self-reproducer occupies a small volume of the space of all possible states of a system, whereby 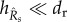, 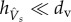. So do generic resources, being a collection of low-entropy, low-entanglement states: therefore, 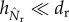, 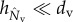. Thus, the condition in (4.1) can be met by many unitaries because
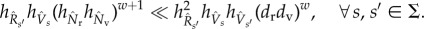
Each unitary permitting self-reproduction is the serial composition of one implementing the *copy phase* and one implementing the *construction phase*. Without loss of generality, I adopt a qubit model, where the replicator consists of *r* qubits and the vehicle of *v* qubits. The information variable *Σ_b_* representing the elementary replicator units comprises, say, the *z*-component eigenvectors of a single qubit. I model generic resources as a fixed input state from *N*, say the simultaneous +1-eigenvector of the *z*-components of the qubits in 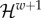 (having the desired features of low-entropy and no-entanglement).

The *copy* unitary **C** (replicating the recipe) must satisfy
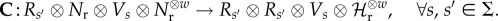


This is realized as a sequence of CNOT*_i_*_,*j*_ from qubit *i* in the parent's replicator subspace 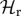 to the qubit *j* of the new instance's performed in the presence of any vehicle:
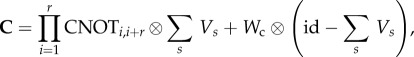
where the unitary *W*_c_ occurs if no vehicle is available. (A possible error-correction process, controlled by the program, is not modelled here.)

The *construction* unitary **B** must satisfy
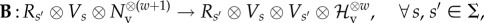
which can be realized by the unitary
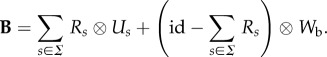
Here, any vehicle executes the recipe *R_s_* to construct a new vehicle *V_s_*, via 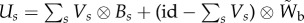 where the unitary *B_s_* is the vehicle construction (possibly including error-correction): 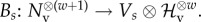 The arbitrary unitaries 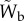 and *W*_b_ express, respectively, the output in the absence of a vehicle and of a program for the vehicle.

Under quantum theory *U_s_* is decomposable into non-specific elementary operations, conditioned on groups of qubits in the replicator. Thus, it does not require the design of *V_s_* being encoded in the dynamical laws. In addition, (by universality) a decomposition into elementary (coherent) quantum gates is allowed. Whether quantum coherence could actually be used, either in living or in artificial self-reproducers, e.g. to enhance the construction efficiency, is an open question in quantum biology [37]. But I have just shown that this possibility is *allowed*. Hence self-reproduction is compatible with quantum theory, as promised.

## 5. Conclusion

I have proved that the physical processes that neo-Darwinian evolutionary theory relies upon are possible under no-design laws, provided that the latter permit information media (and enough generic resources). Under such laws, accurate self-reproduction can occur, but only via von Neumann's replicator–vehicle logic; and a high-fidelity replicator requires an accurate self-reproducer. My argument also highlights that all accurate constructors, under such laws, must contain *knowledge*—an abstract constructor—in the form of a recipe, instantiated in a replicator.

I have also extended von Neumann's model of the *logic* of self-reproduction to quantum theory. This informs further investigations of quantum effects in natural and artificial self-reproducers. Constructor theory has also expressed exactly within fundamental physics the logic of self-reproduction, replication, and natural selection, and the appearance of design. This has promise for a deep unification in our understanding of life and physics.

## Endnotes

1 The issue of what *should* or should not be regarded as life is therefore not relevant here.

2 Subtleties about what portion of the genome is a gene and what natural selection is are not relevant for present purposes. It suffices restricting attention to the logic of natural selection *only*.

3 Their differing from generically fine-tuned laws, or *bio-friendly* laws [8], is discussed in §2.

4 That catalysts exist may indicate fine-tuning in the laws of physics, but not fine-tuning *for biological adaptations*, with which we are concerned here.

5 This issue is different from that of the thermodynamic requirements of replication and self-reproduction, addressed in, e.g. [16,17].

6 The model is faithful *only* insofar the *logic* is concerned. Most realistic details of these processes are irrelevant to their logic and shall be neglected.

7 It is a set of objects that can be copied—not necessarily a set of control instructions.

8 Thus, quantum theory is no-design: the initial state of the universe is a uniform, low entropy state; the allowed elementary interactions do not contain the design of adaptations.

9 Subsidiary theories provide the specific measure of accuracy.

10 This is a schematic representation: *P* need not have a linear structure.

11 I do not model details irrelevant to the self-reproduction logic (e.g. DNA semi-conservative replication).

12 Metabolism-first conjectures such as Dyson's, [11], based on evolution of molecules ‘by random drift’, without template replicators, are thus bound by the present argument to rely on elementary operations, compatible with no-design requirements*.*
